# Supplementary figures and images for: Comparative Chloroplast Genomics, Phylogenomics, and Divergence Times of Sassafras (Lauraceae)
Source: Int J Mol Sci. 2025 Jul 30;26(15):7357. doi: 10.3390/ijms26157357 (PMC12347910; doi:10.3390/ijms26157357)

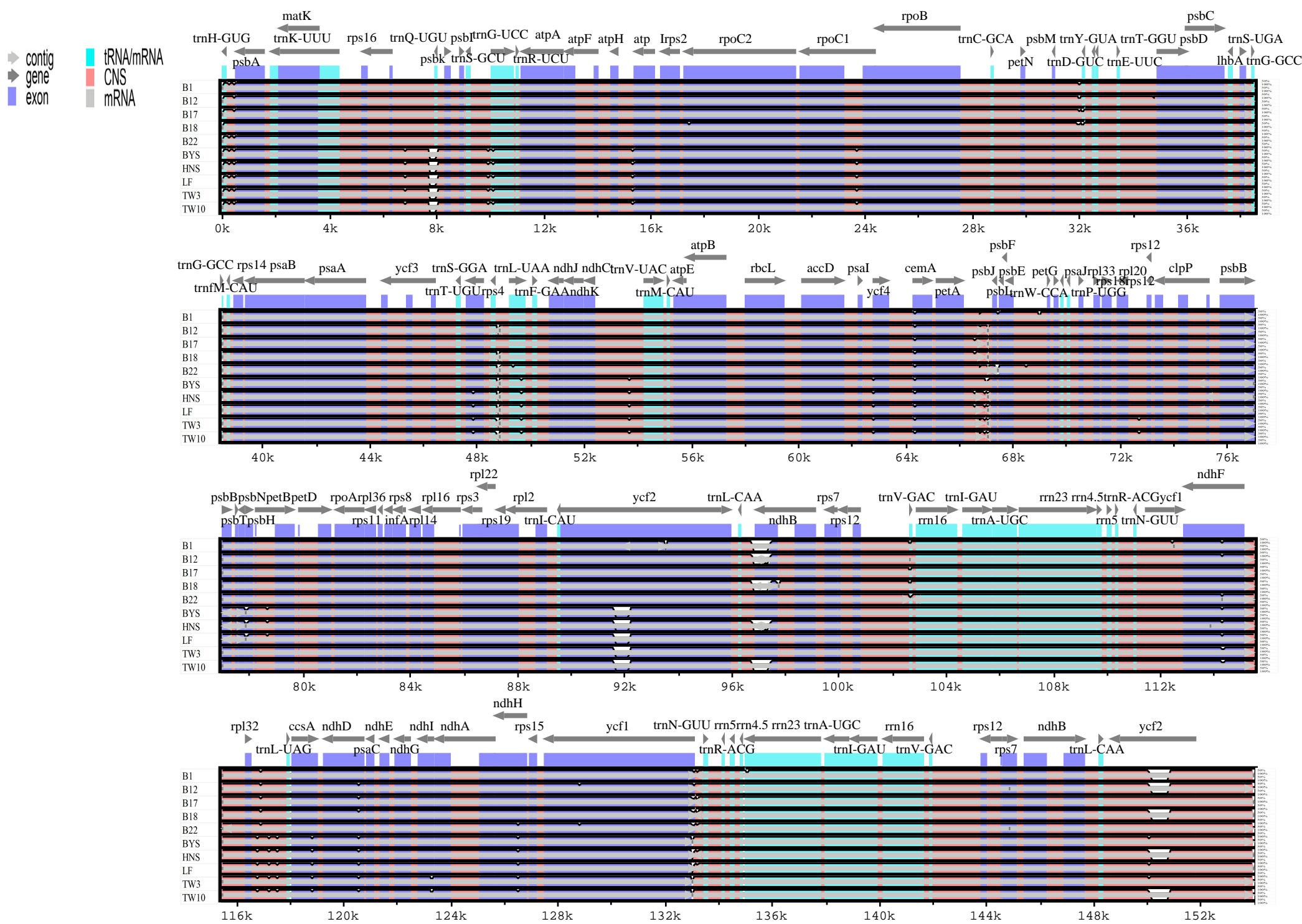

Supplement: Supplementary file 1 [file ijms-26-07357-s001.zip › Figure S1 Comparison of the cp genomes among the 10 Sassafras individuals via mVISTA using annotation of B1 as a reference.pdf]

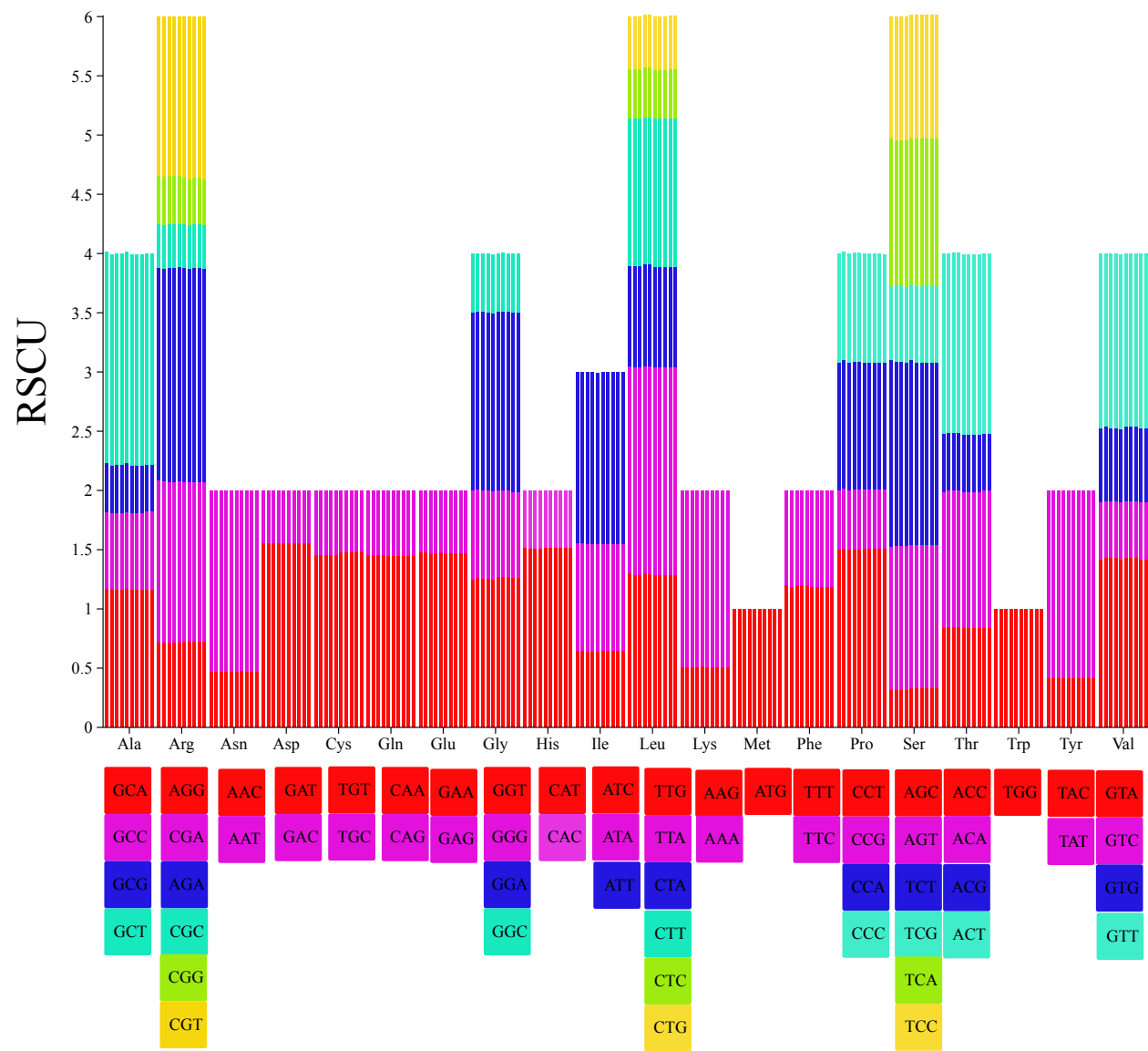

Supplement: Supplementary file 1 [file ijms-26-07357-s001.zip › Figure S2 RSCU analysis of protein coding regions in 10 Sassafras individuals.pdf]

(A)

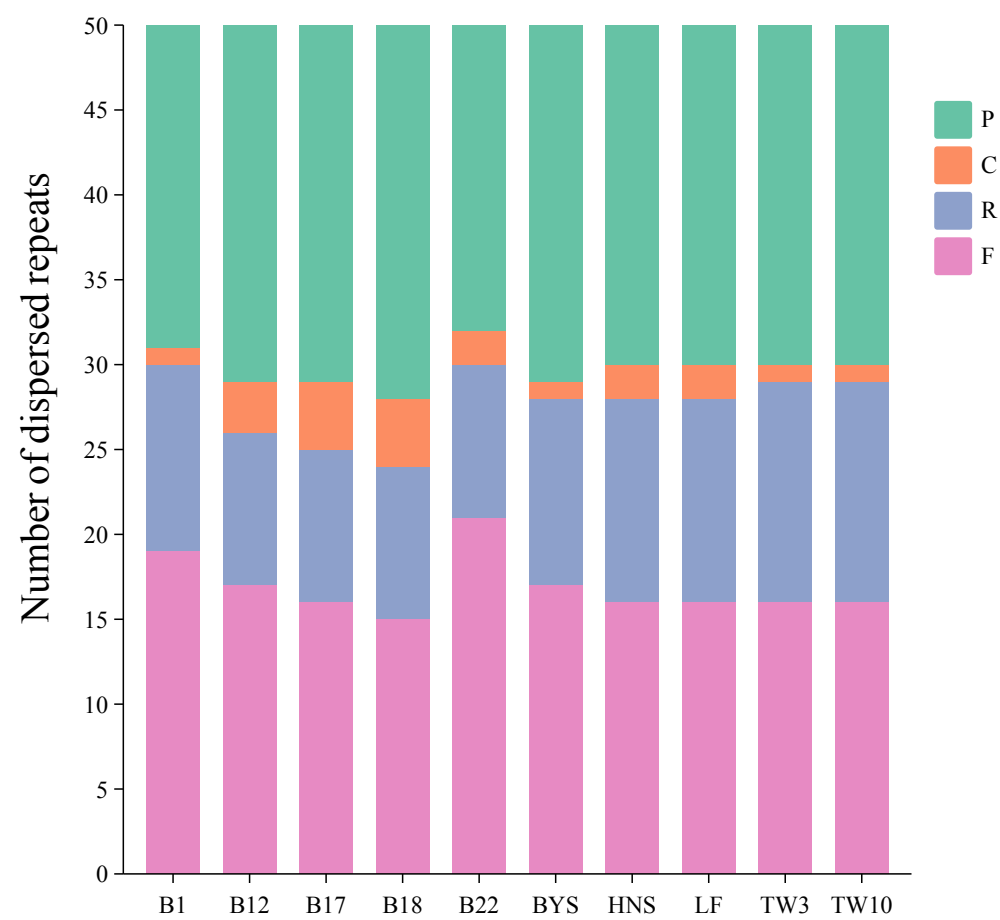

(B)

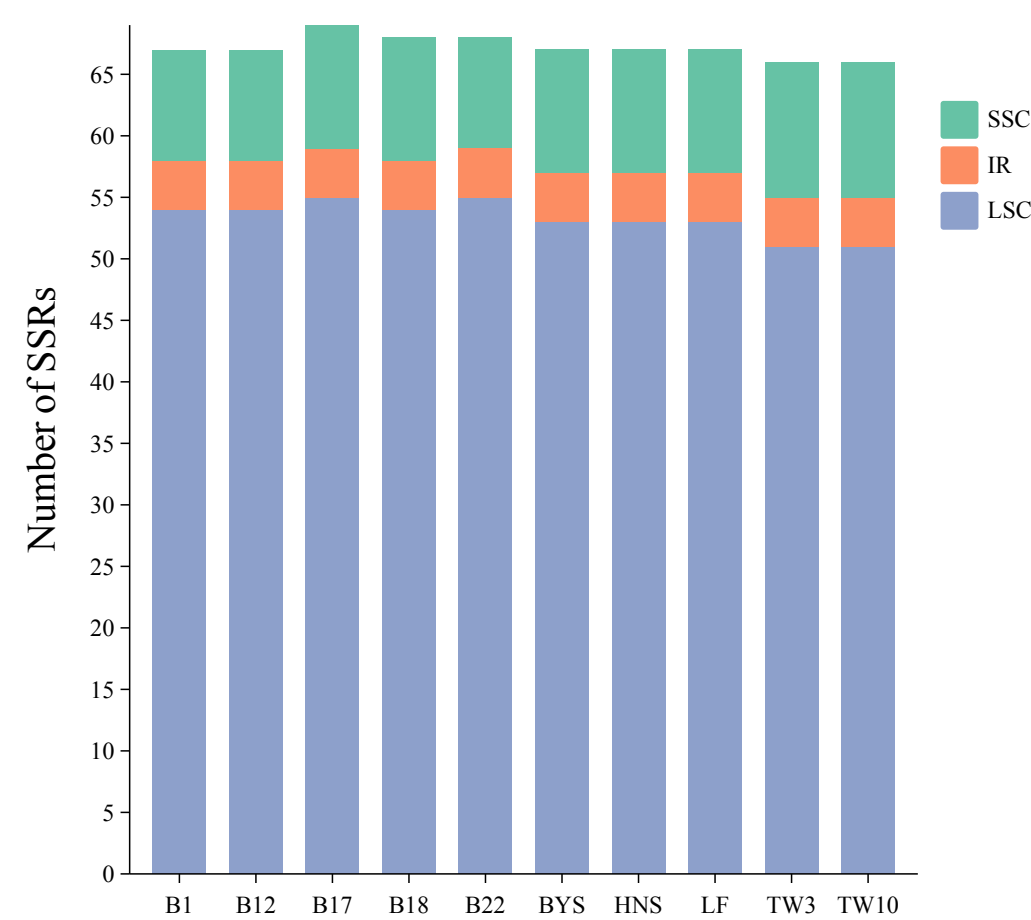

(C)

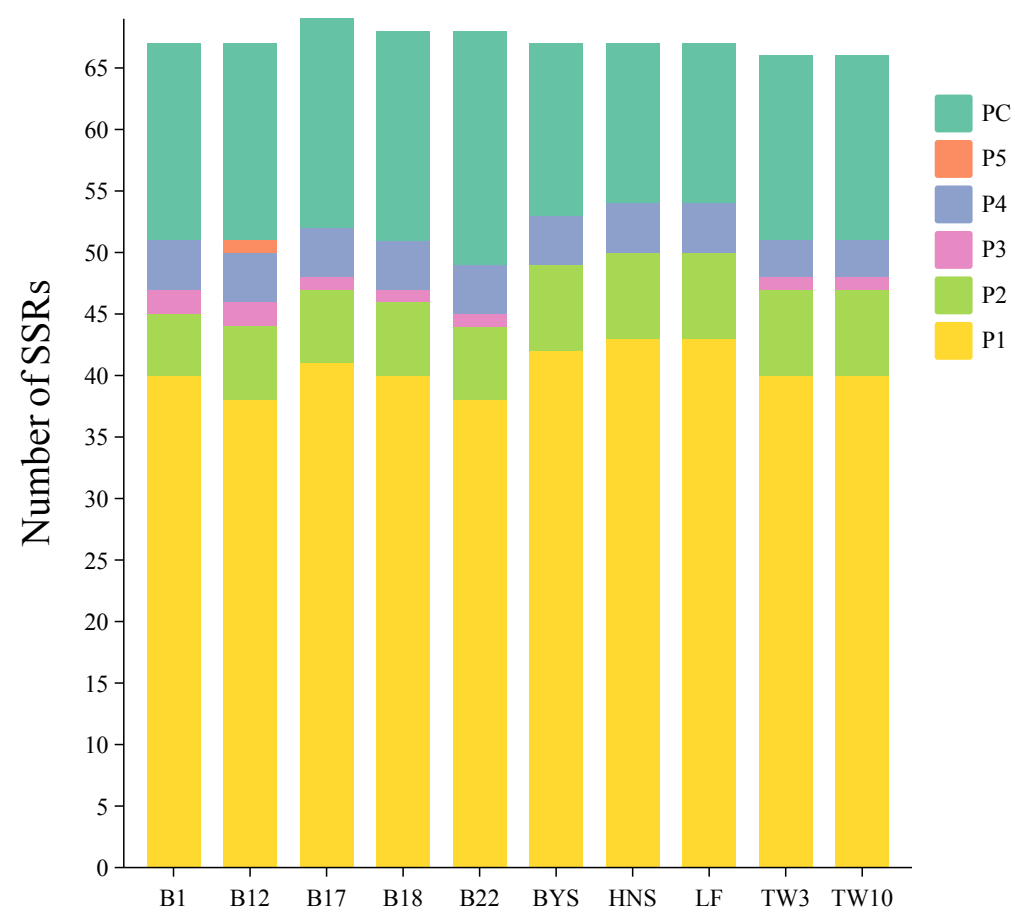

(D)

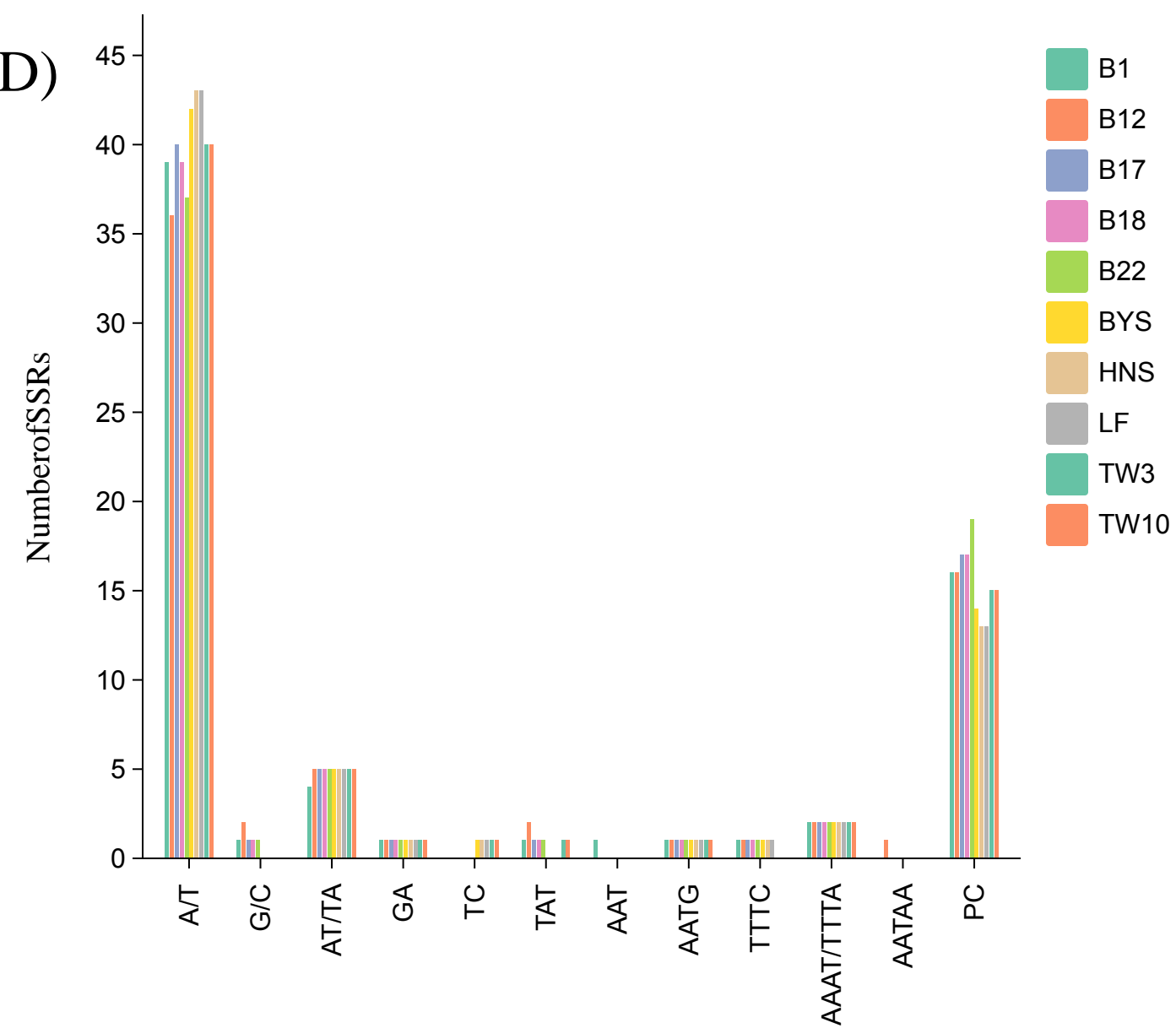

Supplement: Supplementary file 1 [file ijms-26-07357-s001.zip › Figure S3 The number ,type and location of cpSSRs and dispread repeats in 10 Sassafras indi-viduals.pdf]
